# TALEs from a Spring – Superelasticity of Tal Effector Protein Structures

**DOI:** 10.1371/journal.pone.0109919

**Published:** 2014-10-14

**Authors:** Holger Flechsig

**Affiliations:** Department of Mathematical and Life Sciences, Graduate School of Science, Hiroshima University, Higashi-Hiroshima, Japan; University of Zurich, Switzerland

## Abstract

Transcription activator-like effectors (TALEs) are DNA-related proteins that recognise and bind specific target sequences to manipulate gene expression. Recently determined crystal structures show that their common architecture reveals a superhelical overall structure that may undergo drastic conformational changes. To establish a link between structure and dynamics in TALE proteins we have employed coarse-grained elastic-network modelling of currently available structural data and implemented a force-probe setup that allowed us to investigate their mechanical behaviour in computer experiments. Based on the measured force-extension curves we conclude that TALEs exhibit superelastic dynamical properties allowing for large-scale global conformational changes along their helical axis, which represents the *soft* direction in such proteins. For moderate external forcing the TALE models behave like linear springs, obeying Hooke's law, and the investigated structures can be characterised and compared by a corresponding spring constant. We show that conformational flexibility underlying the large-scale motions is not homogeneously distributed over the TALE structure, but instead soft spot residues around which strain is accumulated and which turn out to represent key agents in the transmission of conformational motions are identified. They correspond to the RVD loop residues that have been experimentally determined to play an eminent role in the binding process of target DNA.

## Introduction

TAL (transcription activator-like) effectors are proteins that are secreted in plants by bacteria of the *Xanthomonas* genus. Upon injection into cells they are able to activate transcription of specific target plant genes which may be beneficial for bacterial infection [1],[2]. Hence, a considerable amount of scientific attraction to this protein is owing to its role in the disease of various plant types including important crops as well [3],[4]. Furthermore, artificial TALEs engineered to target prescribed sequences offer interesting applications in genome editing [5]–[7].

The common molecular architecture of TALEs consists of canonical two-helix repeats, each of them involved in the recognition of one specific DNA base, that are arranged around a central axis to form an overall superhelical protein structure that wraps around a central groove in which duplex DNA can be bound [8],[9].

Apparently, adequate understanding of the mechanisms underlying the operation in these important proteins requires a combination of various experimental approaches, including structure determination and biochemical manipulation plus analyses, with detailed methods of molecular modelling. On the other side, the structure-function relationship of proteins, i.e. the principle of how the three dimensional folded protein conformation defines its functional activity, may reveal surprisingly simple patterns. In this regard molecular machines and motors represent a prime example. Their modular architecture, consisting of rigid domains connected by more flexible joints, gives rise to well-organised relative internal motions through which the particular function is implemented [10],[11].

In view of this conception one may ask the seemingly simple question which is probably most appealing from the perspective of a physicist: Given the fact that TALEs look like a spring do they also exhibit spring-like dynamics and what are the benefits of such a structure in terms of its elastic properties? Those questions which refer to the mechanical aspects of the operation of TALE proteins are addressed in this paper.

As we remember from high school physics we can probe the properties of a deformable object by holding it at one end and put a weight on the other end. The force generated by the weight would then induce an extension of that object, i.e. a deviation from its natural length, which can be easily measured. Using a variety of different weights allows to trace the dependence of the extension from the applied force, a relation which is typically used to discuss the objects' elastic properties.

Here, we have performed such experiments in modelling simulations based on the currently available crystal structures of TALEs. For the protein dynamics we have employed the coarse-grained elastic-network description in which the protein is represented as a network of beads connected via deformable strings [12]–[14]. Despite gross simplifications present in such models, they have been proven to perform remarkably well in the prediction of functional chemo-mechanical motions in proteins [15]–[19]. It should be stressed that proteins modelled as elastic networks still present highly complex systems which generally can only be treated numerically and, as shown previously, may exhibit strong nonlinearities in their conformational dynamics [20].

Our analysis was performed for four different TALE structures. We have considered the artificially engineered dHax3 TALE in its free form and in the conformation which was co-crystallized with DNA [8]. Both structures contain 11 TAL repeats which complete one helical turn, but the corresponding pitch is found to be substantially different. Furthermore we have taken into account the structure of the PthXo1 TALE from the rice pathogen *Xanthomonas oryzae*, which was determined in the presence of duplex DNA and reveals an overall two-turn helical shape formed by 23.5 repeats [9]. To allow comparison with dHax3 we have also constructed a shortened one-turn version of PthXo1 (named PthXo1* throughout the paper, see Methods section). All investigated TALE structures are listed in Table 1.

**Table 1:** Investigated Tal effector structures.

TALE	PDB ID	DNA	residues	TALE repeats	immobilized/forced residue	pitch (Å)
dHax3	3V6P	no	Gly^303^-Gly^675^	11 (1b to 12a)	Gly^303^/Gly^675^	60.5
dHax3-DNA	3V6T	yes	Gly^303^-Gly^675^	11 (1b to 12a)	Gly^303^/Gly^675^	35.7
PthXo1	3UGM	yes	Gly^234^-Asp^1032^	23.5 (-1b to 22b)	Gly^234^/Lys^1016^	74.7
PthXo1*	3UGM	yes	Gly^234^-Cys^623^	11.5 (-1b to 10b)	Gly^234^/Gly^608^	36.5

Summary of the TALE structures considered in the force-probe simulations. The pitch was measured as the distance between the alpha-carbon atoms of the immobilised and force residue.

## Methods

We considered the structures of two TALE proteins, that of the artificially engineered dHax3 in its DNA-free form (PDB ID 3V6P) and in a conformation determined in the presence of DNA (3V6T), and that of PthXo1 from the rice pathogen *Xanthomonas oryzae* which was co-crystallized with its DNA target (3UGM). In Table 1 all investigated TALE structures are listed. All figures that display protein conformations in this paper were prepared with the VMD software [21].

### Network construction and dynamics

The elastic network of a TALE protein was obtained by replacing each amino acid of the corresponding structure by a single bead that was placed at the position of the respective alpha-carbon atom (denoted by 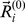 for bead *i*). Then each two beads were connected by a deformable string if their spatial distance was below a prescribed interaction radius *r_int_*. The constructed network of dHax3 consisted of *N* = 373 beads (corresponding to Gly^303^-Gly^675^) and the PthXo1 network had *N* = 789 beads (Gly^234^-Asp^1032^). In the PthXo1* network the shortened structure (Gly^234^-Cys^623^) was considered and *N* = 388 (a summary is given in Table 1). All networks were constructed using an interaction radius of 8 Å.

The total elastic energy of the network 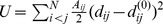 is the sum over all string contributions, where *N* is the number of network beads and *A_ij_* = 1, if beads *i* and *j* are connected by a string, and *A_ij_* = 0 else. Here, 
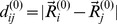
 is the natural length of a string connecting beads *i* and *j* (as extracted from the respective PDB file) and 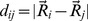 is its corresponding deformed length, with 
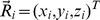
 being the actual position vector of bead *i*. The energy given above represents a rescaled energy in which the dependency from the stiffness constant (which was the same for all strings) was removed. Neglecting thermal fluctuations and hydrodynamical interactions, the dynamics of the network can be described by a set of Newton equations considered in the over-damped limit [17]. For bead *i* the equation of motion is 
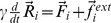
, where 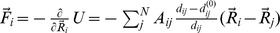 are the internal forces generated by deformed strings which are connected to bead *i* and 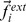 is an external force which can be applied to that bead. Using a rescaled time we can remove the friction coefficient 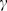 (the same for all beads) on the left hand side of the equations of motion. Due to the energy rescaling the forces have the dimension of lengths in our description. Note that the network dynamics is generally nonlinear since distances are nonlinear functions of the spatial coordinates, i.e. 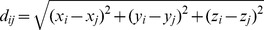. In order to follow the dynamical evolution of the network under external forcing, i.e. to obtain the position of all network beads at every time moment, the equations of motion were numerically integrated. In the simulations we have implemented a first-order integration scheme with a time-step of 0.1.

### Force-probe setup

**Figure 1:**
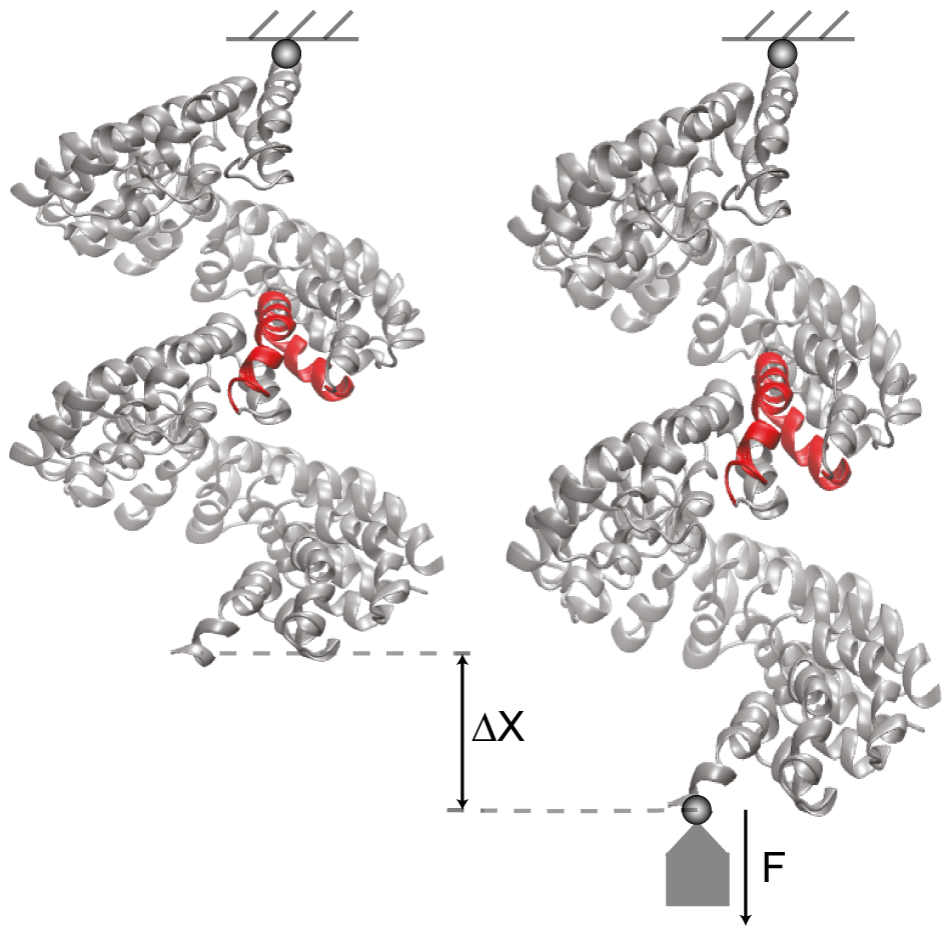
Schematic illustration of the *in silico* force-probe experiments. Exemplarily, the PthXo1 TAL effector is shown in ribbon representation with one end being immobilised and the other end exerted to a force caused by a fictitious weight applied. One selected TAL repeat is shown in red colour.

**Figure 2:**
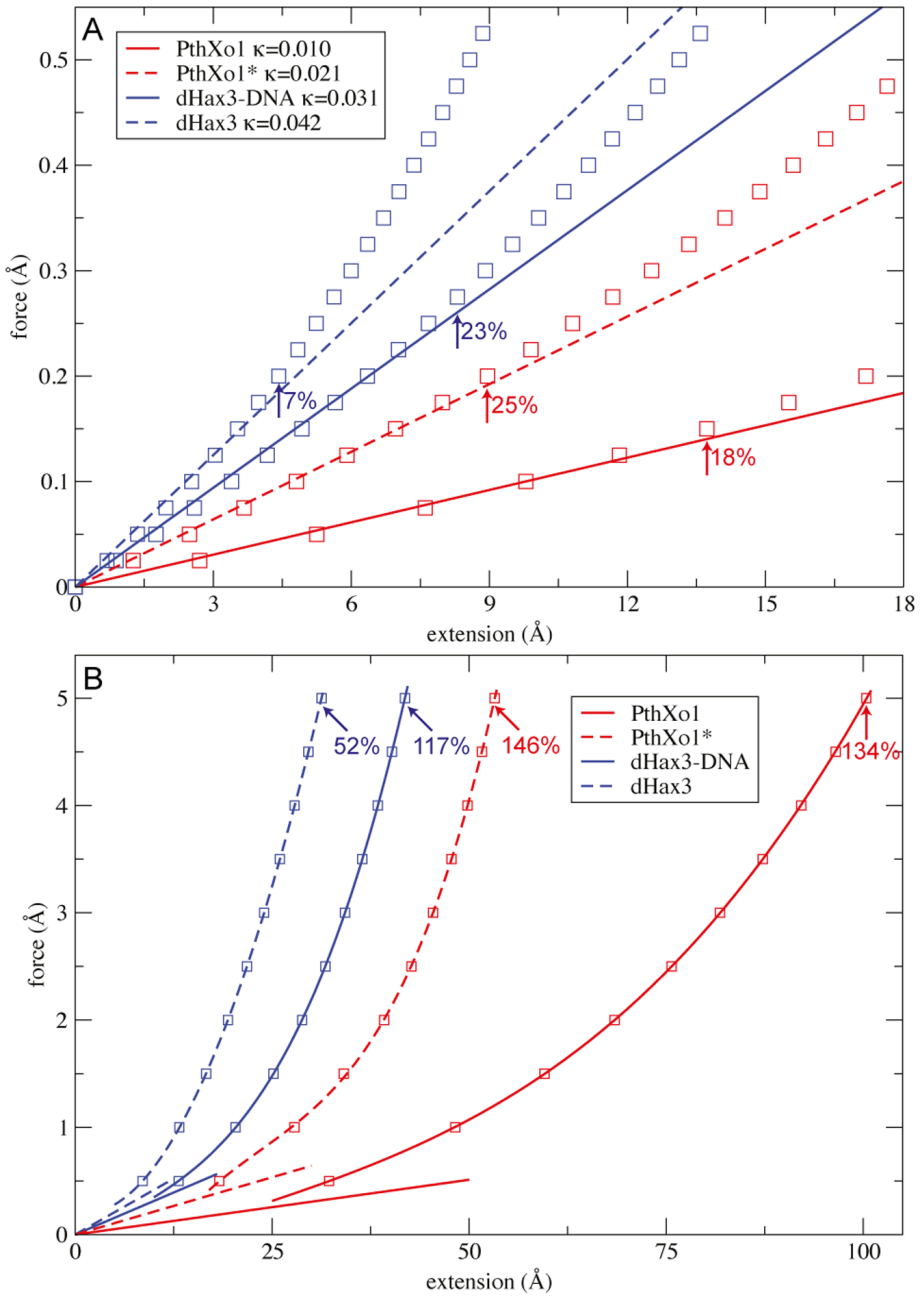
Force-extension relation for the four studied TALEs. A: Linear regression of the data and the derived stiffness constants are shown; the estimated validity range in terms of relative extensions (see Methods) is indicated for each TAL structure. B: Non-linear super-proportional force extension curves for large TALE deformations. For the force of magnitude *F* = 5.0 Å, the relative protein extensions are given.

To implement our force-probe experiment we have immobilised the network at the bead with index 0 (at one side of the protein) and applied an external force to a single bead with index *f* (at the other protein side); see Fig. 1. The force had magnitude *F* and the direction was chosen to coincide with a vector that connects the two selected beads, i.e. 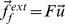 with the unit vector 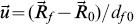. In the simulations we have varied the force magnitude *F* and, each time starting from the initial network, integrated the equations of motions until a steady conformation of the network with the applied force was reached. To detect the steady network state the following condition was implied: The root mean square displacement of the actual network beads with respect to that of the initial network was calculated every 1000th integration step. When the absolute change of two such subsequently determined values was below 0.0001 we have stopped the integration procedure, assuming the network conformation to be sufficiently close to the steady state. In the steady network conformation we have measured the protein extension as 
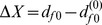
. Relative extensions (given e.g. in Fig. 2) were calculated as 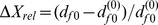.

In the simulations we wanted to stretch the TALE proteins along the ‘spring axis’, i.e. along the superhelical axis which coincides with the orientation of the DNA groove. Therefore, the immobilised network bead (index 0) and the bead to which the force is applied (index f) were carefully chosen. For the dHax3 TALE structures the immobilised bead corresponded to residue Gly^303^ at the beginning of the 1b TALE repeat and the force was applied to the bead which corresponded to residue Gly^675^, which is located at the end of TALE repeat 12a. For PthXo1 and PthXo1* the immobilised bead was Gly^234^ from the long helix of repeat −1. In the case of PthXo1* the forced bead was Gly^608^ from the long helix of repeat 10 and for the entire PthXo1 TALE the forced bead corresponded to residue Lys^1016^ from the long helix of repeat 22 (see summary in Table 1).

**Figure 3:**
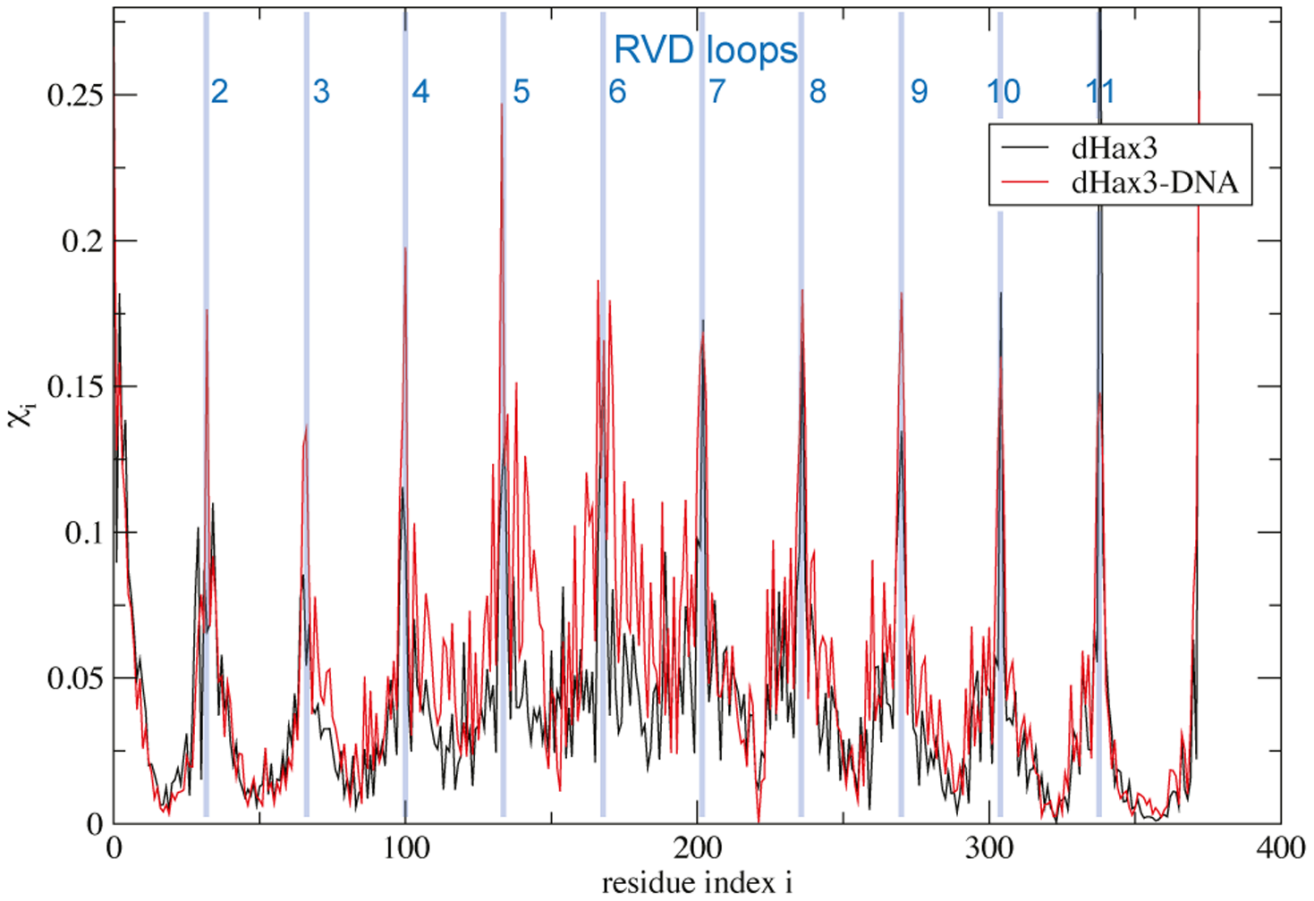
Distribution of local conformational changes in the elongated dHax3 structures obtained after application of an external force with magnitude *F* = 1.0 Å. The deformation value for the residues is plotted (see Methods, lines are used for better visibility). Positions of the RVD loops are indicated by blue lines.

To investigate the distribution of deformations in the final deformed TALE networks (see Fig. 3 and Movie S1), we have assigned each bead *i* the value 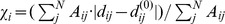, which is the average absolute deformation of strings connected to bead *i*.

### Random forces

A comparison between the dHax3 crystal structures in free and DNA-bound form suggests that the conformational dynamics underlying the functional activity in TALE proteins is dominated by motions along the superhelical axis, and indeed, the setup of our computer experiments was designed to probe the mechanical properties of TALE structures by generating motions in this particular direction. However, in a set of independent additional simulations we aimed to parse the space of generally possible conformational changes of the considered TALE proteins by exerting random external forces to the corresponding elastic networks. The resulting structural changes were then analysed in terms of deformations induced along the superhelical axis and in the lateral direction. Details of these particular simulations can be found in the Text S1.

## Results

A schematic representation of the implemented force-probe setup and the molecular architecture of PtXo1, as an example of the considered TALE structures, is shown in Fig. 1. In the Methods section a detailed description of the performed computer experiments is provided.

The force-extension curves obtained from our simulations are displayed in Fig. 2. They show that through the application of the prescribed forces all four TALE structures were able to undergo significant stretching, revealing that they exhibit an intrinsic flexibility along their superhelical axis. To give an illustration of the performed computer experiments we provide the Movie S1 which shows large-scale global structural changes from a single simulation of force-induced stretching of the PthXo1 structure. Overall we find that three of the TALE proteins could be stretched to far more than twice their initial lengths. The DNA-free dHax3 protein presents an exception; here a force of the same magnitude could induce relative length changes of ‘only’ 52%, i.e. the extended structure is ‘only’ roughly half times longer than the initial one. We have checked whether the identified force-induced strong deformations were reversible and found that in all cases, after the release of the applied force and the immobilisation, the TALE protein returned to its particular initial structure. This finding indicates that the overall structural arrangement of TALEs must possess remarkable elastic properties which may give rise to large-amplitude conformational motions along the superhelical axis.

We observe that the dependence of the extension of TALE structures from the applied force is separated into two regimes. For moderate extensions, i.e. when deviations from the initial length were not too large, a linear relation to the force is found, which represents Hooke's law with the proportionality factor corresponding to the stiffness constant which can be assigned to the respective TALE structure. The validity of this linear behaviour differs for the four TALEs (see Fig. 2); for the dHax3-DNA and PthXo1* the range (as estimated from the force-extension curves) is up to relative extensions of 25%. Across that region and for deformations far beyond the initial protein length we identified a nonlinear regime in which the force grows super-proportional with the extension, i.e. the structure gets stiffer the more it is stretched.

The stiffness constants derived from a linear regression in the linear regime show that the full two-turn PthXo1 TALE is the softest structure as compared to the other TALEs. The single-turn PthXo1* is twice as stiff as PthXo1 but softer than dHax3-DNA by a factor of 1.5. The DNA-free dHax3 protein represents the stiffest of the investigated TALE structures. It should be noted that due to the coarse-grained protein description the absolute scale of the dimensionless stiffness constants is arbitrary.

Apparently, the global large-scale elongations of TALE structures in response to forcing represent a collective effect resulting from the accumulation of local structural deformations which are still small. In the elongated states, as we find, such deformations are inhomogeneously spread over the TALE conformation and multiple residues around which strain is accumulated and which define soft spots of the structure can be identified (results for dHax3 are shown in Fig. 3). They are located along the central DNA binding groove and belong to the short RVD-loops which connect the two alpha-helices of each TAL repeat and are critical for establishing contacts to DNA. Similar observations are made for PthXo1* and full PthXo1 (not shown in Fig. 3 but in the Movie S1 the PthXo1 structure was coloured according to the deformation pattern of its elastic network).

**Figure 4:**
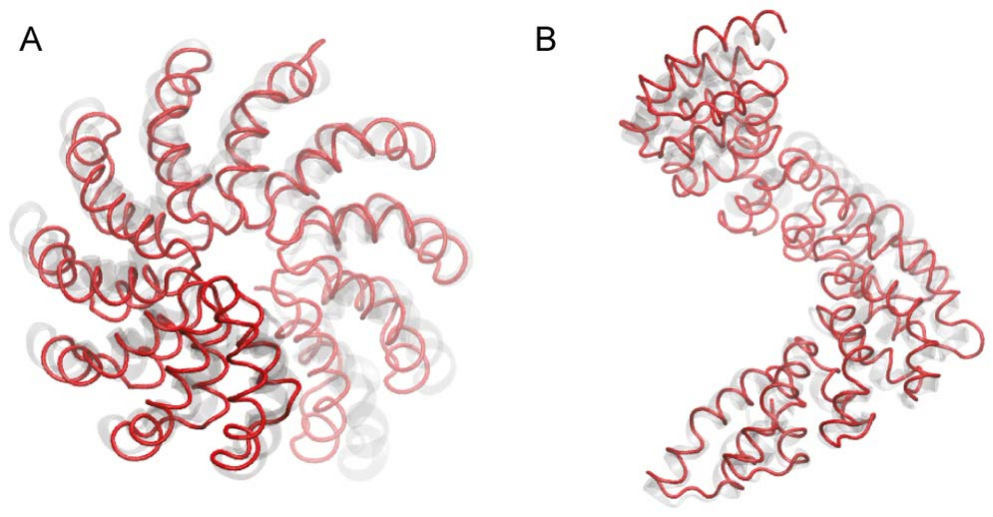
Comparison of dHax3 TALE structures. An *in silico* structure (shown as red tube) obtained from force-induced stretching of the dHax3-DNA conformation is superimposed with the DNA-free crystal structure of dHax3 (shown as transparent grey ribbons). Top view (A) and side view (B) perspective is shown.

We were curious whether conformational motions induced in our simple force-probe setup may cover aspects of the transition between the two dHax3 structures, that determined in the presence of DNA and the DNA-free form. The DNA-bound structure has a length of 35.7 Å (measured as the distance between immobilised and forced residue, which roughly corresponds to the structural pitch, see Methods), whereas the same length of the DNA-free conformation is 60.5 Å. As we see from Fig. 2, the force that would be needed to generate the corresponding extension of 24.8 Å in the dHax3-DNA structure cannot be determined from Hooke's law but instead has to be deduced from the nonlinear relation. From a cubic regression of the dHax3-DNA force-extension data in that regime we computed the corresponding magnitude of the force as 1.45 Å. In a single simulation we have applied this force to the dHax3-DNA elastic network and compared the resulting extended structure with that of the DNA-free dHax3 crystal structure (see Fig. 4). We find that after superposition of their C*α*-atoms they compare with a RMSD-value of 3 Å.

In the main force-probe setup always a single external force was applied to induce extensions of the TALE structures along the superhelical axis. However, to further probe the structural flexibility and test generally possible conformational changes we have performed additional independent simulations employing random external forcing (see Methods). Simulation details are summarised in Text S1 and results are shown in Figure S1. For all considered TALE structures we find that deformations generated along the superhelical direction are generally larger by one order of magnitude as compared to structural changes in the lateral direction (induced by the same random forces). Therefore, our findings evidence that conformational dynamics of the considered TALEs is dominated by motions along the superhelical axis which apparently represent the *soft* direction in such proteins.

## Discussion

In this paper we report results of specific force-probe computer experiments performed for four Tal-effector (TALE) protein structures. Their molecular architecture shows a superhelical arrangements of basic structural repeats, and, crystal structures obtained for a particular TALE have indicated pronounced conformational flexibility along the helical axis [8]. Inspired by their spring-like shape we aimed to investigate whether TALEs also exhibit spring-like dynamical properties. For that purpose we have implemented a very basic setup of computer experiments in which a TALE protein was immobilised at one end and exerted to a force at the opposite site. For the TALE dynamics we have employed the coarse-grained elastic-network description in which the protein is viewed as a meshwork of beads connected via deformable strings. This approach emphasises the mechanical aspects of protein function and allows for an efficient numerical implementation in computer experiments.

Our analysis based on the evaluation of force-extension curves shows that the investigated TALE structures indeed exhibit an enormous flexibility along the superhelical axis and even very large deformations are found to be elastic, i.e. the structural changes are reversible. The dynamical properties of TALEs resemble that of mechanical springs; in particular Hooke's law, i.e. the linear dependence of the extension from the applied force is found to be valid for the considered protein structures. However, there are also differences to ordinary springs. The elastic deformability of a macroscopic mechanical spring, typically designed as a periodically wound metal wire, results solely from its particular helical shape and the spring material is usually stiff in itself. Proteins, however, represent *soft material* and the force-induced global deformations in TALE structures must effectively result from a collective amplification phenomena manifested by the accumulation of local conformational changes. To generate such internal motions requires forces acting on all protein residues and which each have to become larger to induce an overall larger global deformation. While the linear regime is valid for moderate TALE extensions, the external force applied in our simulations, which has to compensate all the internal forces, therefore grows in a super-proportional fashion for larger deformations, meaning that the structures become stiffer the more they are stretched. Similar response curves are well-known for mechanical progressive springs.

The stiffness constants for the four TALE structures obtained from the linear regime resemble what we know from macroscopic springs: A linear spring that is cut in halves can be stretched by only half the fraction by the same force, i.e. it is twice as stiff. When comparing the full PthXo1 TALE, which represents a long spring with two helical turns, with the artificially shortened PthXo1*, which has only one helical turn, we find this behaviour to be reproduced also in the case of TALE protein springs. Comparing the properties of the PthXo1 and dHax3 TALEs is certainly complicated due to the lack of structural data. Although our attempt revealed that PthXo1* has the softer structure, the underlying reasons can only be speculated about. Since both structures comprise 11 TAL repeats per helical turn and the pitch differs only by 0.8 Å, the different elasticity may be owed to the fact that dHax3 is an engineered protein whereas PthXo1 represents a structure evolved under real biological conditions. The dHax3 TALE crystallised in the absence of DNA is found to be the stiffest spring and that for which the validity range of Hooke's law is most narrow. This property can be explained by the fact that this structure, having a pitch of 60.5 Å, already represents an extended spring as compared to its DNA-bound version, which has a much lower pitch of 35.7 Å. Hence, it responds to forcing by a larger stiffness.

Despite its approximate nature, our modelling could establish a link between the structural arrangement of TALE proteins and their dynamical properties and demonstrate that the spring-like shape benefits superelasticity. In our description, amongst other simplifications, we neglected the effect of DNA in the simulations (but we did so for both TALE proteins). It is therefore highly remarkable that our simple setup – i.e. the TALE is hold at one end and a single external force is applied at the opposite end – can reproduce to a decent degree the global structural changes of the DNA-associated transition in the dHax3 TALE. Furthermore our prediction that flexibility is not homogeneously distributed over the TALE structures, but instead soft regions are found along the central DNA binding groove, is consistent with the crystallographic B-factors of the free dHax3 conformation, which are systematically larger (i.e. residues are generally more mobile) for residues from the RVD loops. Our findings suggest that these soft spots represent key agents in the transmission of conformational motions in TALE structures. The enhanced flexibility of RVD loops may be critical for the recognition and binding of bases from the target DNA. Maybe such predictions can be checked in single molecule experiments. Generally it should be possible to probe the flexibility and elastic properties of TALE structures in experiments using e.g. appropriate atomic force microscopy setups.

The super-elastic properties of TALE structures revealed by this study are apparently relevant for the functional activity of these proteins, since they involve the ability to undergo enormous conformational changes along their superhelical axis. Moreover, we have shown that in this direction the TALE structures are *soft* whereas deformations in the lateral orientation, i.e. perpendicular to the superhelical axis, are found to be generally much less pronounced. Indeed, the crystal structures of the artificially engineered dHax3, representing the only TALE for which the DNA-bound and free protein could be determined to date, show that the free conformation is stretched by 70% as compared to the DNA-complexed form, thus providing evidence that the large-scale motions are linked to the interactions between the TALE and DNA. Nuclear magnetic resonance studies together with small-angle X-ray scattering analysis performed for the effector protein PthA are in support of these observations [22] and a recently proposed model based on the analysis of AvrBs3 TALE mutants suggests relative motions of the TALE repeats upon DNA target scanning and in the process of DNA recognition and binding to the RVDs [23]. Furthermore, detailed molecular dynamics simulations of the dHax3 TALE have shown that open-close motions between the two ends of the superhelical structure constitute the dominant conformational dynamics [24]. Hence, from the current experimental and modelling studies the significance of conformational flexibility for binding DNA has emerged as a central aspect of TALE function.

Nonetheless, it should be stressed that in the present situation the knowledge about interactions between TALE proteins and DNA is still confined and functionally relevant mechanism such as recognition and binding of their target sequences are not yet sufficiently explored. In fact, the number of studies devoted to investigate such processes is yet limited and further investigations are needed to elucidate the operation principles of TAL effectors. Modelling studies providing a combination of detailed molecular dynamics descriptions of TALE proteins and their target DNA may indeed play an important role in future studies.

## Supporting Information

Figure S1 **Conformational changes of TALE structures in response to random external forcing.** Each data point corresponds to a single deformed TALE network (50 realisations have been generated for each TALE structure). In such conformations the structural changes as compared to the corresponding original TALE were characterised in terms of the extension along the superhelical axis (shown on the horizontal axis) and that in the lateral direction (shown on the vertical axis). See Text S1 for details.
(TIF)

Text S1 **Details of independent additional simulations performed to probe conformational changes of TALE structures in response to random external forcing.**
(DOCX)

Movie S1 **Force-induced stretching of PthXo1.** Large-amplitude deformations of the PthXo1 TALE in response to external forcing (*F* = 1.0 Å). The colour code represents the distribution of local deformations according to the computed deformation value of each residue in the final steady conformation (see Methods, red colour corresponds to a low value and blue colour to large values).
(MPG)
