# Supplementary figures and images for: TALEs from a Spring – Superelasticity of Tal Effector Protein Structures
Source: PLoS One. 2014 Oct 14;9(10):e109919. doi: 10.1371/journal.pone.0109919 (PMC4196931; doi:10.1371/journal.pone.0109919)

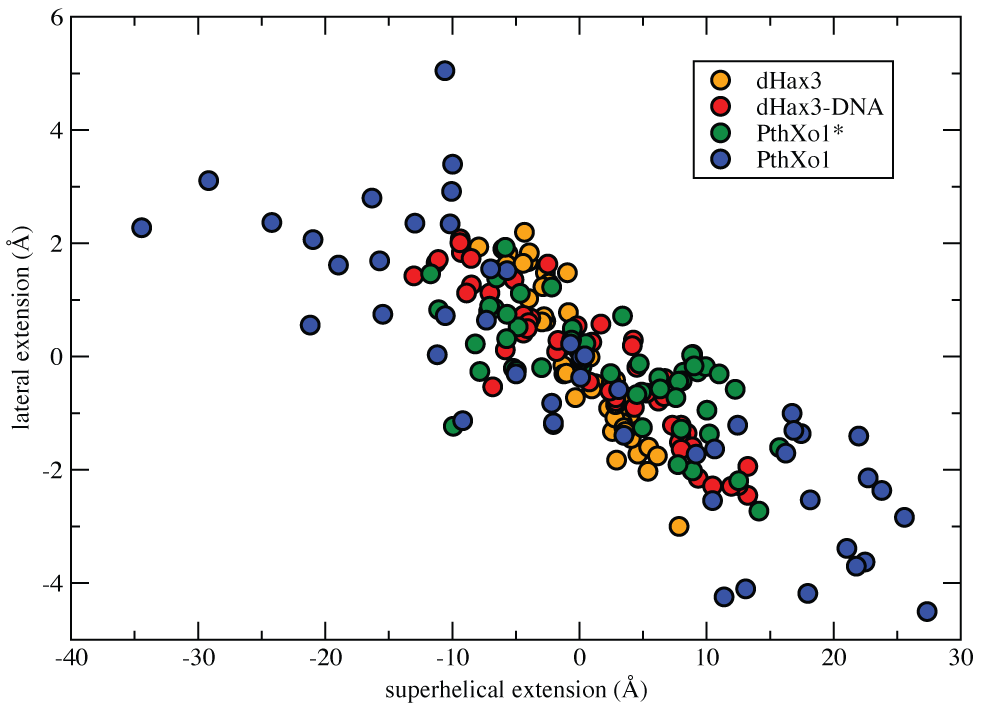

Supplement: Figure S1 — Conformational changes of TALE structures in response to random external forcing. Each data point corresponds to a single deformed TALE network (50 realisations have been generated for each TALE structure). In such conformations the structural changes as compared to the corresponding original TALE were characterised in terms of the extension along the superhelical axis (shown on the horizontal axis) and that in the lateral direction (shown on the vertical axis). See Text S1 for details. (TIF) [file pone.0109919.s001.tif]
