# Supplementary material for: TALEs from a Spring – Superelasticity of Tal Effector Protein Structures
Source: PLoS One. 2014 Oct 14;9(10):e109919. doi: 10.1371/journal.pone.0109919 (PMC4196931; doi:10.1371/journal.pone.0109919)
Supplement: Text S1 — Details of independent additional simulations performed to probe conformational changes of TALE structures in response to random external forcing. (DOCX) [file pone.0109919.s002.docx]

**Text S1: Details of independent additional simulations performed to probe conformational changes of TALE structures in response to random external forcing.**

In order to probe conformational flexibility of TALEs we have generated a set of 50 different deformed structures for each of the four TALE proteins and analyzed the conformational changes in such structures in terms of extensions along the superhelical axis and in the lateral direction. For a particular TALE structure, each single global deformation was obtained by applying random static forces to all beads of its corresponding elastic network. Specifically, we have generated static external forces $\vec{f}_{i}^{ext}$ by choosing the components $f_{i,x},f_{i,y},f_{i,z}$ as random numbers from the interval -1 and +1. Then, we have rescaled the forces such that the normalization condition ${(\sum_{i} \left| \vec{f}_{i}^{ext} \right|^{2})}^{\frac{1}{2}}=F$, with a prescribed value of $F=1.0 Å$, was always fulfilled. In order to obtain the coordinates of the deformed TALE network structure, we have numerically integrated the equations of motions (see Methods section in the main text) in the presence of the external forces until a steady state was reached. To detect the steady state we have computed the distance $d_{f0}$ every 100th integration step. If the absolute change of two subsequently determined distance values was below 0.00001 we have stopped the integration. To prepare the set of 50 deformed structures the entire procedure was repeated, each time starting from the initial unperturbed TALE network but with a different realization of random external forces.

To analyze the global structure changes in response to the external forcing we have determined two values for each deformed TALE structure which allowed us to characterize the difference in the deformed conformations as compared to the corresponding initial unperturbed TALE network. First we have measured the extension along the superhelical axis as $\Delta X=d_{f0}-d_{f0}^{(0)}$, following the same method used for the main force-probe TALE setup. Second we wanted to estimate the extension of TALE in the lateral direction, i.e. perpendicular to the superhelical axis, using a single parameter. To do this we have introduced an axis in the superhelical direction, which was defined by the positions of the network beads $\vec{R}_{0}$ and $\vec{R}_{f}$ (the same that were used to determine the extension along the superhelical axis). Then we measured the shortest distance of each network bead to that axis and averaged over all these values. The difference between this quantity and the same quantity determined for the corresponding unperturbed TALE was used to quantify the lateral extension of the deformed TALE structures. Results of the additional simulations are shown in Fig. S1.
